# Amikacin Suppresses Human Breast Cancer Cell MDA-MB-231 Migration and Invasion

**DOI:** 10.3390/toxics8040108

**Published:** 2020-11-20

**Authors:** Yun-Hsin Wang, Yau-Hung Chen, Wen-Hao Shen

**Affiliations:** 1 Division of Basic Research, Koo Foundation Sun Yat-Sen Cancer Center, Taipei 112, Taiwan; wenhaoshen@gmail.com; 2 Department of Chemistry, Tamkang University, Tamsui, New Taipei City 251, Taiwan; yauhung@mail.tku.edu.tw

**Keywords:** amikacin, TXNIP, siRNA, breast cancer cells

## Abstract

(1) Background: Amikacin is an aminoglycoside antibiotic used for treating gram-negative bacterial infections in cancer patients. In this study, our aims are to investigate the migratory inhibition effects of amikacin in human MDA-MB-231 cells. (2) Methods: We used a wound-healing assay, trans-well analysis, Western blotting, immunostaining and siRNA knockdown approaches to investigate how amikacin influenced MDA-MB-231 cell migration and invasion. (3) Results: Wound healing showed that the MDA-MB-231 cell migration rates decreased to 44.4% in the presence of amikacin. Trans-well analysis showed that amikacin treatment led to invasion inhibition. Western blotting demonstrated that amikacin induced thioredoxin-interacting protein (TXNIP) up-regulation. TXNIP was knocked down using siRNA in MDA-MB-231 cell. Using immunostaining analysis, we found that inhibition of TXNIP expression led to MDA-MB-231 pseudopodia extension; however, amikacin treatment attenuated the cell extension formation. (4) Conclusions: We observed inhibition of migration and invasion in MDA-MB-231 cells treated with amikacin. This suggests inhibition might be mediated by up-regulation of TXNIP.

## 1. Introduction

Metastatic disease is the main cause of mortality in patients with advanced breast cancer. Until recently, breast cancer prevalence rates have remained at a high level. According to public data from the Center for Disease Control and Prevention (CDC), incidence of breast cancer ranges from 196,600 to 245,299, and prevalence rates are 134.8–124.2 per 100,000 women (data were collected between 1999 and 2016) [1]. Breast cancers are categorized into several molecular subtypes based on their expression profile (e.g., estrogen/progesterone receptors positive (ER+/PR+), HER2+ and triple negative). Among them, triple-negative breast cancer is considered to be the most malignant (high probability of visceral metastasis) and difficult to treat [2]. Triple-negative breast cancers do not express either estrogen or progesterone receptors, and there is no over-expression of HER2 protein. The main treatment option for triple negative breast cancer is limited to chemotherapy, since hormonal therapy and anti-HER2 therapeutic regimens cannot be applied [2]. Aside from adverse side-effects, the overall response rate of patients to these cytotoxic reagents is far from satisfactory. Thus, there is an urgent need to develop more effective, safer, and less-toxic anti-cancer drugs.

It is well known that thioredoxin (TRX) and its endogenous inhibitor, thioredoxin-interacting protein (TXNIP), help sustain the cellular reduction/oxidation balance in response to various stresses, and both play a crucial role in cell proliferation, migration and growth [3,4]. For example, it has been reported that TXNIP can increase cell apoptosis and inhibit proliferation, migration and invasion in cancer cells such as non-small cell lung cancer**,** liver cancer, pancreatic cancer and triple-negative breast cancer [5,6,7,8,9]. In this regard, TXNIP might be a potential target for anti-cancer drug development.

Antibiotics are not only well-known agents for treating bacterial infection, they can also be used for cancer treatment. For example, it was reported that salinomycin can inhibit prostate cancer cell growth and migration by reducing the expression of certain oncogenes. Moreover, this antibiotic can selectively kill breast cancer stem cells [10,11,12,13,14]. Through the inhibition of mTOR and surviving pathways [15], Rapamycin was reported to be able to inhibit the proliferation and invasive ability of human gastric cancer cells, while also being able to induce apoptosis. The utilization of antibiotics as an anti-cancer agent is highlighted by these observations, providing a thoroughly researched safety profile. Amikacin is an aminoglycoside antibiotic that is commonly used to treat bacterial infections [16]. In cancer patients, amikacin is often used to treat gram-negative bacterial infections. However, some studies found that certain aminoglycoside drugs might have the potential to be used for cancer treatment [17,18,19]. In this study, our aims were to investigate the inhibitory effects of amikacin in MDA-MB-231 (triple-negative breast cancer cells). We used wound-healing and trans-well analysis to study the cell migratory and invasion activities. Subsequently, we used Western blotting and immunostaining to further evaluate the molecular mechanisms underlying amikacin-induced inhibitory effects.

## 2. Materials and Methods

### 2.1. Cell Culture and Amikacin Treatment

Human breast cancer cell line MDA-MB-231 (1 × 10^4^ cells/plate) was grown in LDF basal nutrient medium (DMEM and 15% Han’s F12 medium) containing 180 mg/L sodium bicarbonate, 15 mM HEPES supplemented with 5% (*v*/*v*) heat-inactivated fetal bovine serum (FBS) and 50 μg/mL of each antibiotic (penicillin and streptomycin) (complete medium). For amikacin treatment (amikacin (25 mg/mL, as stock concentration) was bought from Tai Yu Pharmaceutical Co., Hsinchu, Taiwan), FBS-containing medium was removed and was replaced with a 1% amikacin (250 μg/mL, as working concentration) serum-free medium, incubated at 37 °C for 18 h.

### 2.2. Wound-Healing Assay

The effect of amikacin (1%; 250 μg/mL) on MDA-MB-231 cell migration was studied using a wound healing assay. Cells were seeded and plated on 6-well plates for 18 h. Then, a wound was scratched with wound scratcher and amikacin was added immediately after wound scratching. Each sample was monitored with Incucyte Live-Cell Imaging System and software (Essen Instruments, Ann Arbor, MI, USA). Wound closure was observed every hour for 24 h (photos were taken from each plate at 0, 8 and 24 h) by comparing the mean relative wound density of three independent replicates in each experiment.

### 2.3. Invasion Ability Assay

The cell invasion assay was performed using modified Boyden chamber methods according to the manufacturer’s instructions (BD Biosciences, San Jose, CA, USA). In brief, cells were plated on the inserts at a density of 2 × 10^5^ cells and the cells on the outer surface were harvested and counted after 24 h. Afterwards, the cells in the upper surface of the membrane were removed and invasive cells located between the Matrigel (0.6 mg/mL) to the lower surface of the membrane were fixed with methanol and stained with DAPI. The invasive cells on the lower surface of the membrane were counted with a Leica fluorescent microscope. For data collection, three independent experiments were performed in duplicate.

### 2.4. Western Blotting

The lysate of MDA-MB-231 cells was extracted from the vehicle control (0 ppm of amikacin exposure) or from the cell sample with 250 μg/mL (1%) amikacin exposure using a commercial kit according to the manufacturer’s instructions (Protein Extraction Kit, Millipore, Billerica, MA, USA). Western blotting followed standard procedures except for the use of antibodies against beta-catenin (1:1000), E-cadherin (1:1000), TXNIP (MBL K0205-3, 1:1000) and GAPDH (loading control; 1:5000, LabFrontier LF-PA0018), which were used as the primary antibodies. Images were quantified by using the commercial software ImageQuant TL (Cytiva, Marlborough, MA, USA).

### 2.5. Transfection and siRNA Knockdown

siRNA clone containing TXNIP (pLKOAS3W-TXNIP; Clone ID: TRCN0000059059, NM ID: NM_006472) was obtained from the National RNAi Core Facility (Academia Sinica, Taipei, Taiwan), and was used for the TXNIP knockdown experiment. For the transfection experiment, MDA-MB-231 cells (1 × 10^5^) were seeded in each well and cultured for 24 h at 37 °C. The cells were then co-incubated with 10 µL Lipofectamine 2000 (Thermo Fisher Scientific, Inc., Waltham, MA, USA) and 4 µg plasmids at 37 °C for 6 h. Subsequently, the cells were treated with new medium and incubated at 37 °C for 24 h. Following the transfection procedure, the cells were used for the further experiments.

### 2.6. Antibody Labeling and Images

Cells were seeded on pre-coating collagen slides in a 24-well plate and incubated at 37 °C for overnight culture. Then, the cells were washed twice with phosphate-buffered saline (PBS, pH 7.4) and fixed with PBS containing 4% paraformaldehyde for 12 min at 25 °C. After fixation, the solution was removed by three washes with PBS, and was permeabilized by 0.1% Triton X-100/PBS treatment at 25 °C for 1 h. After rehydration, the cells were treated with 4% bovine serum albumin/PBS and subjected to immunofluorescence staining. To detect beta-catenin protein, mouse antibodies specific to beta-catenin (1:50, R&D) were used as the primary antibodies, and FITC-labeled anti-mouse IgG (1:200, Vector) was used as the secondary antibody. To detect F-actin, anti-F-actin Alexa Fluor 568 was used (1:100, Vector). Immunostained cells were washed thoroughly with PBS. To stain nuclei, fixed cells were incubated with DAPI at 25 °C for 15 min. All samples were observed under a microscope (DM 2500, Leica) equipped with a fluorescent module with FITC, DsRed and DAPI filter cubes (Kramer Scientific, Amesbury, MA, USA). Images were captured by a digital camera (Canon, Taipei, Taiwan) and migrating cells were filmed.

### 2.7. Statistical Analysis

The data were expressed as averages ± SD and tested by Microsoft Excel TTEST. *p* < 0.05 was identified as statistically significant.

## 3. Results

### 3.1. The Effects of Amikacin on MDA-MB-231 Cell Proliferation and Migration

**Figure 1:**
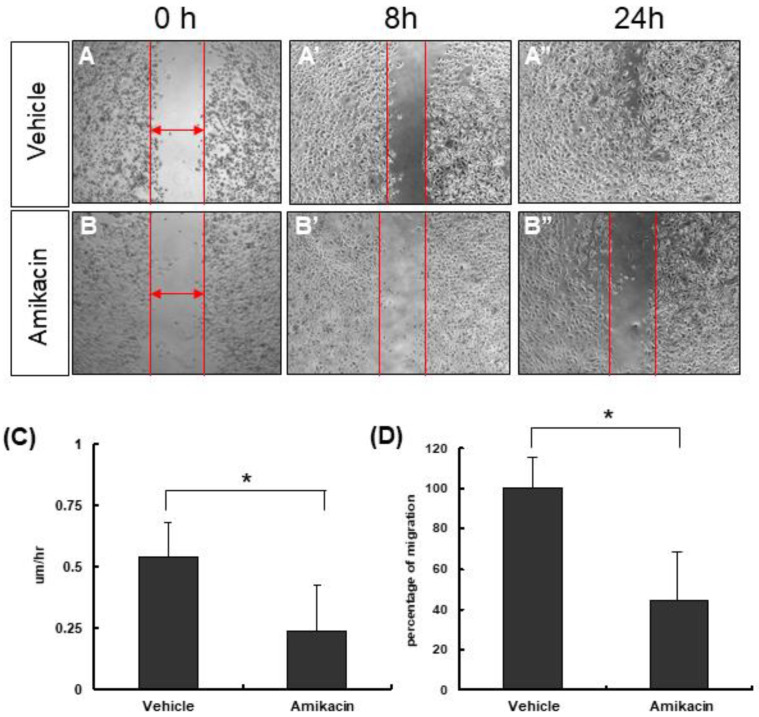
Effects of amikacin on cell migration. (**A**,**A’**,**A’’**) MDA-MB-231 cells were treated with PBS (vehicle) or (**B**,**B’**,**B’’**) amikacin for 0, 8 and 24 h. (**C**) Cell migration rates (μm/h) of both the vehicle- and amikacin-treated groups. (**D**) Percentages of cell migrations for both vehicle- and amikacin-treated groups. Values are means of three independent experiments (*N* = 3), with standard deviations represented by vertical bars. Migration rates and percentage of migration from three independent experiments were measured and statistically analyzed. (Averages ± SD; * *p* < 0.05).

First of all, we used an MTT assay to evaluate the effects of amikacin on MDA-MB-231 cell proliferation. Our results showed that amikacin treatment increased the cell viability to 1.6~2.2 fold in either complete medium or serum-free medium, suggesting that amikacin has no cell proliferation inhibition ability (Supplemental Materials Figure S1). Next, we carried out the wound-healing assay to test the effects of amikacin on cell migration ability. We created a 500-μm wound, took photos and measured the width of each group at different time points (0 and 8 h). As shown in Figure 1, the migration rates by 8 h were 0.54 ± 0.14 μm/h (averages ± SD, *N* = 3) for the vehicle group (PBS) and 0.24 ± 0.18 μm/h (averages ± SD, *N* = 3) for amikacin-treated group. After normalization (supposing that the migration percentage for the vehicle group was 100%), the migration percentage of the amikacin-treated group was reduced to 44.4%.

### 3.2. Amikacin Inhibits MDA-MB-231 Cell Invasion

**Figure 2:**
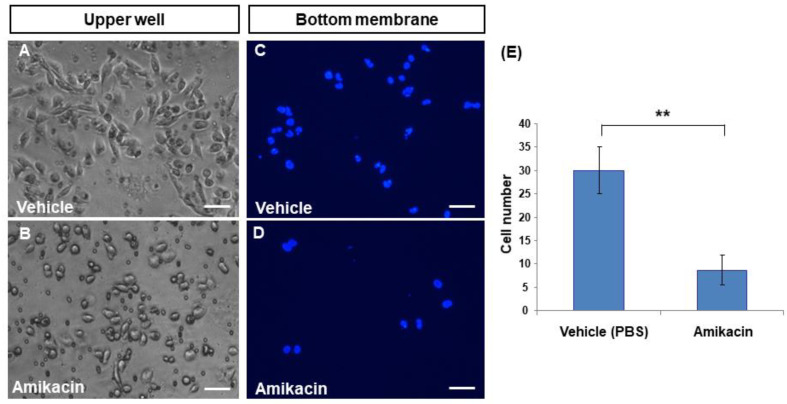
Effects of amikacin on cell invasion. Trans-well analysis of both vehicle- (**A**,**C**) and amikacin-treated (**B**,**D**) MDA-MB-231 cells. Photos were taken from the upper wells (bright field, **A**,**B**) or from the bottom (stained with DAPI, dark field, **C**,**D**). (**E**) Number of invasion cells of both vehicle- and amikacin-treated groups. Values are the means of three independent experiments (for additional pictures, see Supplemental Materials Figure S2), with standard deviations represented by vertical bars. Number of invasion cells from three independent experiments were measured and statistically analyzed. (Averages ± SD; ** *p* < 0.01). Scale bar: 25 μm.

To further demonstrate whether amikacin is able to inhibit cell invasion, we performed trans-well analysis. In the untreated group, MDA-MB-231 cells produced a spindle-like shape (Figure 2A) in the upper well. In contrast, amikacin-treated MDA-MB-231 cells had a roundish shape (Figure 2B), suggesting that amikacin might be able to affect cytoskeleton organization. To calculate how many cells reached the bottom membranes, members were stained with DAPI fluorescent dye. Results showed that 30 ± 8.7 and 5 ± 3.2 cells migrated to the bottom membrane for the vehicle- and amikacin-treated groups, respectively (Figure 2C–E). On the basis of these observations, we theorize that amikacin treatment is able to reduce the invasive potential of MDA-MB-231 cells.

### 3.3. Amikacin Treatment Leads to Up-Regulation of TXNIP

**Figure 3:**
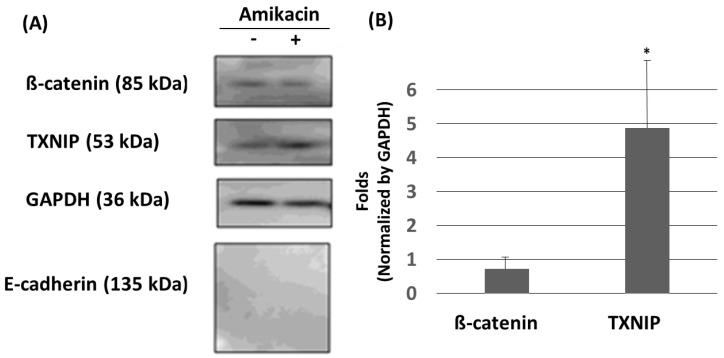
Effects of amikacin on MDA-MB-231 cell protein expressions. (**A**) Western blotting showing the expression of ß-catenin, TXNIP, GAPDH and E-cadherin in MDA-MB-231 cells treated with or without amikacin for 24 h. (**B**) Western blotting signals were quantified by using the “1D gel analysis” function of the commercial software ImageQuant TL. Data were collected from three independent experiments, normalized by the expressions of GAPDH, and statistically analyzed. (Averages ± SD; * *p* < 0.05).

Cell migration abilities are delicately controlled by many positive or negative factors. For example, E-cadherin (a key component of the adherents’ junctions) and thioredoxin-interacting protein (TXNIP) are usually considered to be negative regulators, but ß-catenin might have dual roles [6,14,20,21,22,23]. In this study, we carried out a Western blot experiment to investigate whether E-cadherin, ß-catenin and TXNIP would be affected by amikacin. As shown in Figure 3, ß-catenin and TXNIP expressions were detected, but E-cadherin was undetectable in the vehicle group. A previous study showed that E-cadherin was undetectable in MDA-MB-231 cells [24], which agrees with our observation. In the amikacin-treated group, the expressions of E-cadherin were still undetectable. The expressions of ß-catenin were slightly down-regulated to 0.72 ± 0.34 fold (averages ± SD, *N* = 3, at 24 h, *p* > 0.1) but the expressions of TXNIP were up-regulated to 4.87 ± 2.02 fold (averages ± SD, *N* = 3, at 24 h, *p* < 0.05) (Figure 3). These observations suggest that (i) ß-catenin and TXNIP might play a role in amikacin-induced cell migration and invasion; (ii) amikacin treatment was unable to induce E-cadherin in MDA-MB-231 cells.

### 3.4. Knockdown of TXNIP Affects Cell Morphology

**Figure 4:**
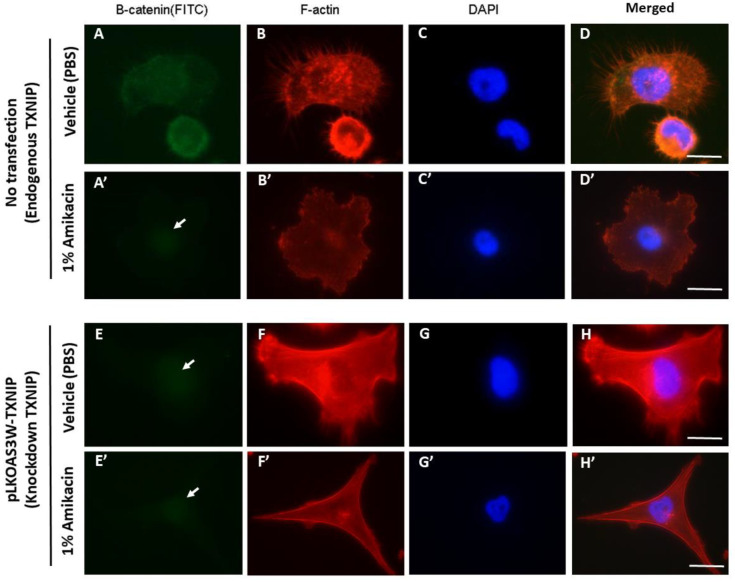
Antibody labeling of ß-catenin and F-actin in MDA-MB-231 cells and TXNIP knockdown MDA-MB-231 cells. Fluorescent microscopy of ß-catenin and F-actin distributions in vehicle treatments (**A**–**H**) and 1% amikacin treatments (**A’**–**H’**) in either MDA-MB-231 cells (**A**–**D**,**A’**–**D’**) or TXNIP knockdown MDA-MB-231 cells (**E**–**H**,**E’**–**H’**). ß-catenin distribution is reported in green (white arrows indicate the very faint signals in **A’**,**E**,**E’**), F-actin expression pattern is reported in red and DAPI nuclei labeling is shown in blue. (**D**,**D’**,**H**,**H’**) are merged figures. Scale bar: 10 μm.

Finally, we used an siRNA knockdown approach to decrease endogenous TXNIP expressions in MDA-MB-231 cells, followed by an antibody staining experiment (counterstained with DAPI to make the nucleus visible), and tried to uncover the relationships among amikacin, ß-catenin and TXNIP during cell migration and the invasion process. Results showed that endogenous ß-catenin and TXNIP expressed and distributed evenly throughout the whole cell (Figure 4A–C). In contrast, in the amikacin-treated group, endogenous ß-catenin expressions were nearly undetectable in the cytoplasm, with only very faint signals observed in the nucleus (Figure 4A’, arrow indicates). F-actin staining showed that amikacin treatment led to less filament actin polymerization and distribution at the edges of cells (Figure 4B,B’).

Next, we used knockdown endogenous TXNIP expressions to further examine the effects of amikacin on cell migration and invasion. In the TXNIP knockdown cells, ß-catenin expressions were extremely faint in both the vehicle (PBS-treated) and amikacin-treated groups (Figure 4E,E’). Results from F-actin staining showed that amikacin treatment decreased filament actin polymerization and attenuated MDA-MB-231 cells’ pseudopodia-like structure extensions compared to the vehicle control (PBS-treated) (Figure 4F,F’).

## 4. Discussion

The results of this study show that amikacin treatment suppresses the migration and invasion of MDA-MB-231 cells. As with other antibiotics, prodigiosin was reported to be able to inhibit proliferation, migration and invasion of nasopharyngeal carcinoma cells [25]. In non-tumor cells, tobramycin was demonstrated to be able to affect corneal epithelial cell migration [26], and cefazolin was shown to inhibit human mesenchymal stromal cell (hMSC) migration to wound-healing sites [27]. In particular, amikacin was able to affect bull spermatozoal motility and mouse embryonic stem cell migration ability [28,29,30]. These observations show that certain antibiotics might have the potential to be applied for cancer treatment or wound healing in corneal or bone trauma. Our results of F-actin staining showed that amikacin treatment attenuated MDA-MB-231 (TXNIP knockdown) cells’ pseudopodia-like structure extensions (Figure 4), suggesting that cytoskeleton organization affected by amikacin treatment might be partially achieved through TXNIP up-regulation. A previous study showed that one of the approaches for antibiotics to inhibit bacteria growth is by inhibiting the bacteria cytoskeleton’s polymerization [30]. These observations suggest that a cytoskeleton might be a putative target for antibiotics.

From a molecular perspective, our data indicate that the expressions of TXNIP was up-regulated after amikacin treatment. Similar observations have been reported in previous studies. For example, the antitumor activity of mitomycin C and dorzolamide was mediated by stimulating the expression of TXNIP [31]. Co-treatment with polymethoxyflavonoid and tunicamycin led to up-regulation of TXNIP in human neuroblastoma cells [32]. These observations highlight the importance of how antibiotics affect TXNIP expressions in tumor cells.

As for other antibiotics, quinolone derivatives possess promising anti-cancer activity, and some of them have already been approved to treat cancers or for use under clinical trials. For example, quinolone can be used as an AXL kinase inhibitor, which are involved in tumor cell growth [33,34]; Harmol has been used to induce cell death of human non-small cell lung cancer A549 cells [35]; nifuroxazide treatment induces apoptosis, and inhibits cell migration and invasion in osteosarcoma [36]; and Brefeldin A can regulate Bip/Akt-related autophagy in colorectal cancer cells [37]. Together with our findings, we propose that antibiotics might have the potential to develop as anti-tumor drugs.

## 5. Conclusions

In conclusion, we observed the inhibition of migration and invasion in MDA-MB-231 cells treated by amikacin. We suggest that this inhibition might be mediated by the up-regulation of TXNIP.

## Supplementary Materials

The following are available online at https://www.mdpi.com/2305-6304/8/4/108/s1, Figure S1: The cell survival rates were calculated with or without amikacin treatment by MTT assay. Figure S2: The cell morphology in the upperwell after amikacin treatment by transwell migration assay.
