# Supplementary material for: Amikacin Suppresses Human Breast Cancer Cell MDA-MB-231 Migration and Invasion
Source: Toxics. 2020 Nov 20;8(4):108. doi: 10.3390/toxics8040108 (PMC7712503; doi:10.3390/toxics8040108)
Supplement: Supplementary file 1 [file toxics-08-00108-s001.pdf]

Supplementary Materials: Amikacin Suppresses Human Breast Cancer Cell MDA-MB-231 Migration and Invasion

Yun-Hsin Wang, Yau-Hung Chen and Wen-Hao Shen

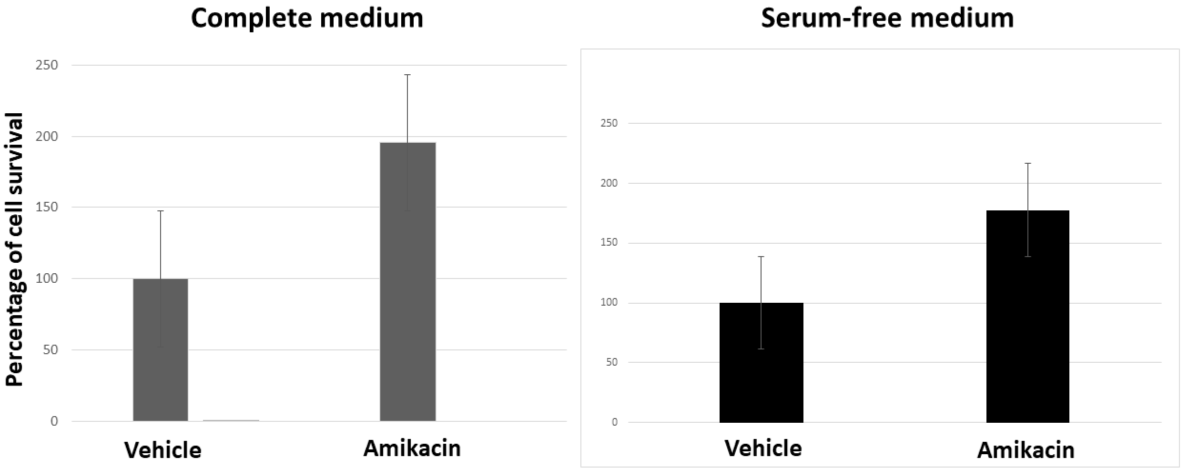

Figure S1. The cell survival rates were calculated with or without amikacin treatment by MTT assay. All data were collected from three independent experiments and statistically analyzed by Excel software (averages ± SD; * $p < 0.05$).

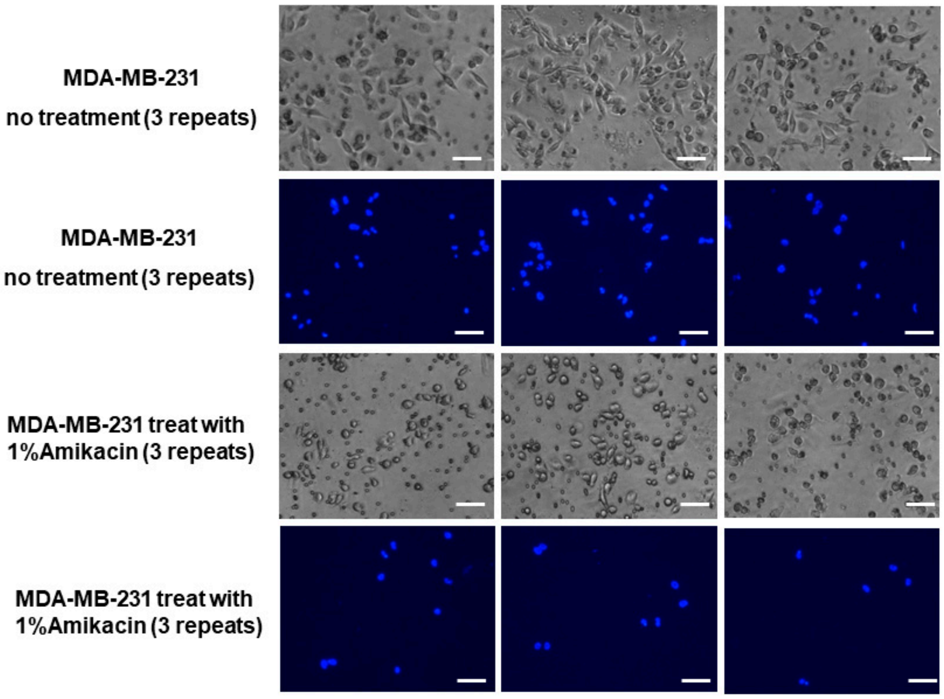

Figure S2. The cell morphology in the upperwell after amikacin treatment by transwell migration assay. Photos were taken from three independent experiments (with or without 1% amikacin treatment). Scale bar: 25 μm.
